# Associations of serum 25-hydroxyvitamin D and vitamin D receptor polymorphisms with dengue severity in hospitalized Vietnamese children

**DOI:** 10.1016/j.ijregi.2026.100989

**Published:** 2026-08-30

**Authors:** Phan Viet Hung, Nguyen Minh Phuong, Nguyen Thi Cam Tien, Tran Thi Huynh Nhu, Pham Minh Quan, Truong Van Dat, Nguyen Thi Thuy Duong, Thai Minh Dien, Trinh Nhut Truong, Hua Kim Chi, Bui Kim Dang, Vo Van Thi

**Affiliations:** 1 Faculty of Medicine, Can Tho University of Medicine and Pharmacy, Can Tho City, Vietnam; 2 Ca Mau Obstetrics and Pediatrics Hospital, Ca Mau City, Vietnam; 3 Faculty of Medicine, Nam Can Tho University, Can Tho City, Vietnam

**Keywords:** 25-Hydroxyvitamin D, Vitamin D receptor, VDR polymorphisms, Dengue severity, Children, Vietnam

## Abstract

**Objectives:**

To investigate the associations of serum 25-hydroxyvitamin D (25(OH)D) concentrations and vitamin D receptor (VDR) polymorphisms with dengue severity in hospitalized Vietnamese children.

**Methods:**

This cross-sectional study included 190 children aged ≤16 years with laboratory-confirmed dengue admitted to Ca Mau Obstetrics and Pediatrics Hospital, Vietnam. Disease severity was classified according to the World Health Organization 2009 criteria. Serum 25(OH)D concentrations were measured, and four VDR polymorphisms (FokI, BsmI, ApaI, and TaqI) were genotyped. Associations with severe dengue were assessed using univariable and multivariable logistic regression.

**Results:**

Thirty-two children (16.8%) had severe dengue. Median serum 25(OH)D concentrations were lower in children with severe dengue than in those with non-severe dengue (21.26 vs 26.65 ng/mL; *P* = 0.002). Higher serum 25(OH)D concentration remained associated with lower odds of severe dengue after adjustment for age group, sex, and IgG seropositivity (adjusted odds ratio = 0.921 per 1 ng/mL increase; 95% confidence interval, 0.855-0.992; *P* = 0.029). No significant associations were detected between the four VDR polymorphisms and dengue severity.

**Conclusion:**

Lower serum 25(OH)D concentrations were associated with severe dengue, whereas the investigated VDR polymorphisms were not. Prospective multicenter studies are needed to clarify temporality and causality.

## Highlights

• Severe dengue was associated with lower serum 25-hydroxyvitamin D concentrations.
• Higher 25-hydroxyvitamin D remained associated with lower severe-dengue odds after adjustment.
• No significant association was found between vitamin D receptor variants and dengue severity.
• Findings provide pediatric dengue evidence from southern Vietnam.

## Introduction

Dengue is a rapidly expanding global health threat, with increasing geographic spread and disease burden over recent decades [1]. Children are particularly vulnerable to severe manifestations, including dengue hemorrhagic fever and dengue shock syndrome, which are associated with substantial morbidity and mortality [1,2]. Disease severity reflects complex interactions among viral factors, host immunity, and prior infection history, but the immunological mechanisms underlying severe dengue remain incompletely understood [[3], [4], [5]].

Vitamin D is increasingly recognized as an immunomodulatory hormone in addition to its role in calcium homeostasis. Through the vitamin D receptor (VDR), its active metabolite regulates innate and adaptive immune responses, including antimicrobial peptide production, cytokine signaling, and T-cell differentiation [[6], [7], [8], [9]]. The VDR is expressed in monocytes, macrophages, dendritic cells, and T lymphocytes. Activation of the vitamin D–VDR pathway may suppress pro-inflammatory cytokines, promote anti-inflammatory responses, and enhance production of antimicrobial peptides such as cathelicidin and defensins [[7], [8], [9]]. These effects are relevant to dengue, in which excessive immune activation, endothelial dysfunction, vascular permeability, and plasma leakage contribute to severe disease [5,10,11].

Previous studies evaluating the association between serum 25-hydroxyvitamin D (25(OH)D) concentrations and dengue severity have reported inconsistent findings. Some studies found lower 25(OH)D concentrations in severe dengue, whereas others found no significant association. Genetic variation in the VDR gene may also influence vitamin D signaling and host immune responses. Four commonly studied single-nucleotide polymorphisms—FokI (rs2228570), BsmI (rs1544410), ApaI (rs7975232), and TaqI (rs731236)—have been associated with altered receptor function and susceptibility to infectious diseases, but their role in dengue remains unclear.

Vietnam is hyper-endemic for dengue, and the Mekong Delta carries a substantial disease burden. However, data on serum vitamin D status and VDR polymorphisms in Vietnamese children with dengue are limited, and few pediatric studies have evaluated both circulating 25(OH)D concentrations and major VDR polymorphisms simultaneously. Therefore, this study investigated the associations of serum 25(OH)D concentrations and VDR polymorphisms with dengue severity in hospitalized Vietnamese children.

## Methods

### Study design and population

A cross-sectional analytical study was conducted at Ca Mau Obstetrics and Pediatrics Hospital, Ca Mau Province, southern Vietnam. Participant recruitment, data collection, and biological sample collection were conducted from November 16, 2024, to January 2026. Children aged ≤16 years who were admitted during this period and had laboratory-confirmed dengue infection were eligible for inclusion. Dengue infection was confirmed by nonstructural protein 1 (NS1) antigen testing, serological testing, and/or reverse transcription-polymerase chain reaction (RT-PCR), in accordance with national and institutional guidelines.

To minimize selection bias, eligible participants were enrolled consecutively. Dengue severity was classified according to the World Health Organization (WHO) 2009 guidelines [12]. Patients were categorized as having either non-severe dengue (dengue without warning signs and dengue with warning signs) or severe dengue, defined by severe plasma leakage, severe bleeding, or severe organ impairment. This binary classification served as the primary study outcome. Exclusion criteria included pre-existing liver disease, chronic kidney disease, immunodeficiency, or receipt of vitamin D supplementation within the preceding 3 months.

A total of 190 children with complete clinical and biochemical data were included in the overall analysis. Genotyping data were available for 184 participants for FokI, 187 for BsmI, and 168 for both ApaI and TaqI. Because this was an observational study conducted within a predefined recruitment period, all eligible children admitted during the study period were enrolled. The final sample size was therefore determined by the number of eligible children meeting the inclusion criteria. The sample size allowed evaluation of associations between serum vitamin D levels and dengue severity; however, it may have been insufficient to detect modest genetic effects related to VDR polymorphisms.

### Clinical and laboratory data

Demographic data, including age and sex, were collected at enrollment. Laboratory investigations included serial measurements of platelet count, hematocrit, and white blood cell count during hospitalization. For analysis, the lowest platelet count (nadir) and the highest hematocrit values recorded during hospitalization were used. Dengue diagnostic test results, including NS1 antigen, immunoglobulin M (IgM), immunoglobulin G (IgG), and RT-PCR, were recorded from admission samples. Dengue virus serotyping was performed by RT-PCR on admission samples.

### Measurement of serum 25(OH)D

Serum 25(OH)D concentrations were measured from blood samples collected during the critical phase of dengue infection (typically between days 3 and 6 of illness) using a chemiluminescent immunoassay on the iFlash 1800 automated immunoassay analyzer (YHLO Biotech Co., Ltd., Shenzhen, China), according to the manufacturer’s instructions. Internal quality control procedures were performed routinely in accordance with laboratory standards. Vitamin D deficiency was defined as serum 25(OH)D concentrations <20 ng/mL, insufficiency as 20-29 ng/mL, and sufficiency as ≥30 ng/mL, in accordance with the Endocrine Society clinical practice guidelines [13].

### Genotyping of VDR polymorphisms

Genomic DNA was extracted from peripheral blood samples using standard extraction procedures. Four common VDR polymorphisms, FokI (rs2228570), ApaI (rs7975232), TaqI (rs731236), and BsmI (rs1544410) were genotyped using PCR amplification of the target regions followed by direct Sanger sequencing. All genotyping analyses were performed at the Center for Molecular Biomedicine, University of Medicine and Pharmacy at Ho Chi Minh City, Vietnam.

### Statistical analysis

Categorical variables were summarized as frequencies and percentages, whereas continuous variables were presented as medians with interquartile ranges. Comparisons between children with non-severe dengue and severe dengue were performed using Pearson’s chi-square test or Fisher’s exact test for categorical variables, as appropriate.

Serum 25(OH)D concentrations were compared between severity groups using the Mann–Whitney U test. Vitamin D status was categorized as deficient (<20 ng/mL), insufficient (20-29 ng/mL), and sufficient (≥30 ng/mL), and differences in distribution between groups were assessed using the chi-square test.

For genetic analyses, genotype frequencies of the four VDR polymorphisms (FokI, BsmI, ApaI, and TaqI) were compared between severity groups using Pearson’s chi-square test or Fisher’s exact test, as appropriate. Hardy-Weinberg equilibrium was assessed for each polymorphism using the chi-square goodness-of-fit test.

The relationship between serum 25(OH)D concentrations and laboratory parameters was evaluated using Spearman’s rank correlation analysis. Correlation coefficients (*r*) and corresponding *P*-values were reported.

To evaluate factors associated with severe dengue and account for measured potential confounding, univariable and multivariable logistic regression analyses were performed. Variables with a *P*-value < 0.20 in univariable analysis were considered for inclusion in the multivariable model. The final model included age group, sex, IgG seropositivity, and serum 25(OH)D concentration. Given the limited number of severe dengue events (*n* = 32), the number of covariates was restricted to reduce the risk of model overfitting. Results are presented as crude odds ratios (ORs) and adjusted odds ratios (aORs) with 95% confidence intervals (CIs). Serum 25(OH)D concentration was entered as a continuous variable.

No data imputation was performed. Clinical and biochemical analyses were based on complete cases (*n* = 190), whereas genotype analyses used available cases for each polymorphism: FokI, *n* = 184; BsmI, *n* = 187; and ApaI and TaqI, *n* = 168.

All statistical analyses were performed using IBM SPSS Statistics version 27.0 (IBM Corp., Armonk, NY, USA). All tests were two-sided, and a *P*-value <0.05 was considered statistically significant.

## Results

### Study population and baseline characteristics

Among 190 children with laboratory-confirmed dengue, 158 (83.2%) had non-severe dengue and 32 (16.8%) had severe dengue. Children aged 6-16 years accounted for 81.1% of the cohort and were more frequent in the severe group than in the non-severe group (96.9% vs 77.8%; *P* = 0.012). Sex distribution did not differ significantly between groups (*P* = 0.119).

NS1 antigen and RT-PCR were positive in 86.3% and 81.1% of participants, respectively. IgG seropositivity was more frequent in the severe group (25.0% vs 10.8%; *P* = 0.044), whereas NS1 antigen, IgM, and RT-PCR positivity did not differ significantly between groups (Table 1).

**Table 1:** Baseline characteristics of study participants.

Characteristic	Total (n = 190)	Non-severe (n = 158)	Severe (n = 32)	*P*-value
**Age group, n (%)**				0.012
0-5 years	36 (18.9)	35 (22.2)	1 (3.1)	
6-16 years	154 (81.1)	123 (77.8)	31 (96.9)	
**Sex, n (%)**				0.119
Male	101 (53.2)	88 (55.7)	13 (40.6)	
Female	89 (46.8)	70 (44.3)	19 (59.4)	
**Diagnostic test, n (%)**				
NS1 antigen (+)	164 (86.3)	137 (86.7)	27 (84.4)	0.778
IgM (+)	26 (13.7)	23 (14.6)	3 (9.4)	0.578
IgG (+)	25 (13.2)	17 (10.8)	8 (25.0)	0.044
RT-PCR (+)	154 (81.1)	126 (79.7)	28 (87.5)	0.307

Data are presented as n (%). *P*-values were calculated using Pearson’s chi-square test or Fisher’s exact test, as appropriate.

Abbreviations: IgG, immunoglobulin G; IgM, immunoglobulin M; NS1, nonstructural protein 1; RT-PCR, reverse transcription-polymerase chain reaction.

### Serum 25(OH)D concentrations and dengue severity

Median serum 25(OH)D concentration was lower in children with severe dengue than in those with non-severe dengue (21.26 [20.39-26.73] vs 26.65 [21.89-30.69] ng/mL; *P* = 0.002). The distribution of vitamin D deficiency, insufficiency, and sufficiency did not differ significantly between groups (*P* = 0.219), although vitamin D sufficiency was less frequent in severe dengue (12.5% vs 26.6%) (Table 2).

**Table 2:** Serum 25(OH)D concentrations and vitamin D status according to dengue severity.

Variable	Non-severe dengue (n = 158)	Severe dengue (n = 32)	*P*
25(OH)D, ng/mL, median (IQR)	26.65 (21.89-30.69)	21.26 (20.39-26.73)	0.002
Deficient (<20 ng/mL)	21 (13.3)	6 (18.8)	0.219
Insufficient (20-29 ng/mL)	95 (60.1)	22 (68.8)	
Sufficient (≥30 ng/mL)	42 (26.6)	4 (12.5)	

Data are presented as n (%) unless otherwise indicated.

Abbreviations: 25(OH)D, 25-hydroxyvitamin D; IQR, interquartile range.

### Correlation between vitamin D levels and laboratory parameters

Serum 25(OH)D concentration correlated positively with minimum platelet count (*r* = 0.201; *P* = 0.005) and negatively with maximum hematocrit (*r* = −0.206; *P* = 0.004). No significant correlation was observed with minimum white blood cell count (*r* = 0.132; *P* = 0.069) (Supplementary Table S1).

### VDR polymorphisms and dengue severity

Genotyping data were available for 184 participants for FokI, 187 for BsmI, and 168 for ApaI and TaqI. FokI and ApaI showed nominal deviations from Hardy-Weinberg equilibrium, whereas BsmI and TaqI did not (Supplementary Table S2). Genotype distributions were not significantly associated with dengue severity for any polymorphism examined (Table 3). Minor allele frequencies were low for BsmI and TaqI.

**Table 3:** Association between VDR polymorphisms and dengue severity.

SNP	Genotype/allele	Non-severe dengue	Severe dengue	*P*-value
*FokI (rs2228570)*	CC	58 (84.1)	11 (15.9)	0.889
	TC	62 (81.6)	14 (18.4)	
	TT	33 (84.6)	6 (15.4)	
	Allele C/T	0.58/0.42	0.58/0.42	
*BsmI (rs1544410)*	GG	135 (81.8)	30 (18.2)	0.378
	GA	20 (90.9)	2 (9.1)	
	AA	0 (0)	0 (0)	
	Allele G/A	0.94/0.06	0.97/0.03	
*ApaI (rs7975232)*	GG	71 (83.5)	14 (16.5)	0.494
	GT	63 (82.9)	13 (17.1)	
	TT	7 (100)	0 (0)	
	Allele G/T	0.73/0.27	0.76/0.24	
*TaqI (rs731236)*	TT	128 (83.1)	26 (16.9)	0.473
	TC	13 (92.9)	1 (7.1)	
	CC	0 (0)	0 (0)	
	Allele T/C	0.95/0.05	0.98/0.02	

Data are presented as n (%) unless otherwise indicated. *P*-values were calculated using Pearson's chi-square test or Fisher's exact test, as appropriate. Genotyping data were available for 184 participants for FokI, 187 for BsmI, and 168 for both ApaI and TaqI. The reported P-values refer to comparisons of genotype distributions between severity groups. Allele frequencies are presented descriptively and were not subjected to separate hypothesis testing.

Abbreviations: SNP, single-nucleotide polymorphism; VDR, vitamin D receptor.

### Serum vitamin D levels according to VDR genotype

Serum 25(OH)D concentrations did not differ significantly across genotype groups for any of the four VDR polymorphisms (Supplementary Table S3). Median serum 25(OH)D concentrations ranged from 23.78 to 27.27 ng/mL across the examined genotypes.

### Logistic regression analysis of factors associated with severe dengue

Univariable logistic regression identified age group, IgG seropositivity, serum 25(OH)D concentration, and sex as variables meeting the prespecified *P* < 0.20 criterion for inclusion in the multivariable model. After adjustment for age group, sex, and IgG seropositivity, higher serum 25(OH)D concentration remained associated with lower odds of severe dengue (adjusted odds ratio = 0.921 per 1 ng/mL increase; 95% confidence interval 0.855-0.992; *P* = 0.029). Specifically, each 1 ng/mL increase in serum 25(OH)D concentration was associated with approximately 7.9% lower odds of severe dengue. Age group, IgG seropositivity, and sex were not statistically associated with severe dengue in the adjusted model (all *P* > 0.05). The complete results of the logistic regression analyses are presented in Table 4.

**Table 4:** Univariable and multivariable logistic regression analyses of factors associated with severe dengue.

Variable	Crude OR (95% CI)	*P*-value	Adjusted OR (95% CI)	*P*-value
Age group (6-16 vs 0-5 years)	8.821 (1.163-66.925)	0.035	5.219 (0.655-41.564)	0.119
IgG seropositivity	2.765 (1.074-7.115)	0.035	2.205 (0.804-6.051)	0.125
Serum 25(OH)D	0.905 (0.844-0.971)	0.005	0.921 (0.855-0.992)	0.029
Sex (female vs male)	1.837 (0.849-3.977)	0.119	1.587 (0.694-3.623)	0.273

Reference categories were age 0-5 years, IgG seronegativity, and male sex. Variables with *P* < 0.20 in univariable analysis were entered into the multivariable model. Bold values indicate *P* < 0.05.

Abbreviations: 25(OH)D, 25-hydroxyvitamin D; CI, confidence interval; IgG, immunoglobulin G; OR, odds ratio.

## Discussion

This cross-sectional study investigated the associations of serum 25(OH)D concentrations and VDR gene polymorphisms with dengue severity among hospitalized Vietnamese children. The principal findings were that serum 25(OH)D concentrations were significantly lower in children with severe dengue, higher serum 25(OH)D concentrations remained associated with lower odds of severe dengue after multivariable adjustment, and serum 25(OH)D concentrations correlated with platelet count and hematocrit. In contrast, none of the four VDR polymorphisms examined was associated with dengue severity or serum 25(OH)D concentrations.

### Study population and baseline characteristics

Among the 190 enrolled children, 32 (16.8%) were classified as having severe dengue according to the WHO 2009 [12] criteria. Older children were disproportionately represented in the severe dengue group, with 96.9% of severe cases occurring among those aged 6-16 years compared with 77.8% of non-severe cases (*P* = 0.012). This finding may reflect the cumulative risk of prior dengue exposure in older children living in endemic regions, thereby increasing the likelihood of secondary infection.

Consistent with this explanation, IgG seropositivity was significantly more frequent among children with severe dengue than among those with non-severe dengue (25.0% vs 10.8%; *P* = 0.044). IgG seropositivity may reflect previous dengue exposure; however, IgG positivity alone does not definitively classify secondary infection [14,15]. During secondary infection with a heterologous dengue serotype, pre-existing non-neutralizing antibodies may facilitate viral entry into Fc receptor-bearing cells, leading to increased viral replication, exaggerated immune activation, endothelial dysfunction, and plasma leakage [16].

Our findings are consistent with previous studies demonstrating an association between secondary dengue infection and disease severity [16]. Several epidemiological and immunological investigations have identified prior dengue exposure as one of the strongest risk factors for severe dengue, particularly among children living in hyper-endemic regions. These observations are particularly relevant in Vietnam, where the co-circulation of multiple dengue virus serotypes increases the likelihood of secondary heterologous infections. Collectively, these findings support the central role of host immune responses, in addition to viral factors, in determining the clinical outcome of dengue infection.

### Serum 25(OH)D concentrations and dengue severity

Children with severe dengue had significantly lower median serum 25(OH)D concentrations than those with non-severe dengue (21.26 vs 26.65 ng/mL; *P* = 0.002). Although the distribution of categorical vitamin D status did not differ significantly between groups, a lower proportion of vitamin D sufficiency was observed among children with severe dengue. A similar inverse association between serum 25(OH)D concentrations and disease severity has also been reported in a Singaporean adult cohort, in which lower vitamin D levels were associated with an increased risk of severe hemorrhagic complications [17]. Together, these findings support the hypothesis that vitamin D deficiency may contribute to immune dysregulation and excessive inflammatory responses, both of which play central roles in the pathogenesis of severe dengue. In contrast, a large-scale study by Villamor et al. [18] (2017) found no significant difference in serum 25(OH)D concentrations across severity groups. Similar observations have been reported in pediatric cohorts from Sri Lanka and Eastern India, where vitamin D deficiency was associated with plasma leakage and severe dengue manifestations [19,20]. These conflicting findings suggest that the observed relationship may be influenced by multiple factors, including geographic setting, population characteristics, circulating serotypes, and the timing of blood sampling during the dynamic course of dengue infection.

The biological plausibility of this association is supported by the immunomodulatory effects of vitamin D. The active metabolite, 1,25-dihydroxyvitamin D, signals through the VDR to suppress pro-inflammatory cytokines, including TNF-α, IL-6, and IL-12, while promoting anti-inflammatory pathways such as IL-10 production [8,9,21]. Because excessive immune activation and endothelial dysfunction contribute to plasma leakage in dengue, vitamin D may help modulate these processes and thereby influence disease severity [22,23]. In addition, vitamin D induces the expression of antimicrobial peptides, including cathelicidin (LL-37) and defensins, which have been reported to exert antiviral effects [7,11,24].

Supporting the clinical relevance of this association, serum 25(OH)D concentrations correlated positively with minimum platelet count (*r* = 0.201; *P* = 0.005) and negatively with maximum hematocrit (*r* = −0.206; *P* = 0.004), two laboratory parameters closely related to plasma leakage and disease severity. Thrombocytopenia and hemoconcentration are hallmark manifestations of severe dengue and reflect increased vascular permeability and plasma leakage. The observed correlations therefore suggest that lower vitamin D concentrations may be associated with more pronounced plasma leakage and hematologic disturbances during acute dengue infection. No significant correlation was observed between serum 25(OH)D concentrations and white blood cell count (*r* = 0.132; *P* = 0.069). Although causality cannot be inferred from this cross-sectional study, these findings support further investigation of the relationship between vitamin D status and pathophysiological features of severe dengue.

### VDR gene polymorphisms and dengue severity

In the present study, none of the four VDR polymorphisms examined, FokI (rs2228570), BsmI (rs1544410), ApaI (rs7975232), and TaqI (rs731236), was significantly associated with dengue severity. Similarly, serum 25(OH)D concentrations did not differ significantly across genotype groups, suggesting that no statistically significant associations were detected with circulating vitamin D status. Furthermore, neither genotype distributions nor allele frequencies differed significantly between children with severe and non-severe dengue, providing additional evidence against a major role of these polymorphisms in determining disease severity.

Although VDR polymorphisms have been implicated in susceptibility and clinical outcomes in several infectious and immune-mediated diseases, evidence regarding their role in dengue remains limited and inconsistent [3,6,25]. Previous studies have reported conflicting findings, with some suggesting associations between specific VDR variants and dengue susceptibility, while others failed to demonstrate relationships with disease severity. In contrast, several studies from dengue-endemic regions have consistently reported associations between lower vitamin D levels and more severe clinical manifestations, suggesting that circulating vitamin D status may be more relevant to disease progression than common inherited variations in the VDR gene [26,27]. The absence of significant associations in our study may indicate that the contribution of VDR genetic variation to dengue severity is modest compared with other host, viral, and environmental factors that influence disease progression [25]. Taken together, these findings suggest that circulating vitamin D status may be more closely associated with dengue severity than common inherited variations in the VDR gene.

A recent Vietnamese pediatric study involving 178 children similarly found that higher serum 25(OH)D concentrations were independently associated with lower odds of severe dengue, whereas FokI, ApaI, TaqI, and BsmI polymorphisms were not associated with disease severity [28]. These findings are consistent with our results and suggest that circulating vitamin D status may be more relevant to dengue severity than common VDR variants.

Several explanations may account for the absence of significant genetic associations in the present study. First, the frequencies of minor alleles were relatively low, particularly for the BsmI and TaqI polymorphisms, and homozygous variant genotypes were not observed for either single-nucleotide polymorphism. Such distributions reduce statistical power and may limit the ability to detect modest genetic effects. Nominal deviations from Hardy-Weinberg equilibrium were observed for FokI and ApaI. These deviations may reflect the limited sample size, missing genotype data, population characteristics, or potential genotyping-related factors and therefore warrant cautious interpretation of the genetic findings. Second, dengue severity is a complex phenotype influenced by multiple host, viral, immunological, and environmental factors. Consequently, the contribution of individual VDR polymorphisms is likely to be modest compared with stronger determinants such as viral characteristics, immune responses, nutritional status, and inflammatory activity during acute infection. In addition, analysis of individual single-nucleotide polymorphisms may not fully capture the functional effects of genetic variation across the VDR locus.

Therefore, while our findings do not support a significant role for the investigated VDR polymorphisms in dengue severity, they do not exclude the involvement of the vitamin D signaling pathway in dengue pathogenesis. Future studies with larger sample sizes and haplotype-based approaches may provide a more comprehensive assessment of the role of VDR genetic variation in dengue pathogenesis [6].

### Multivariable analysis: factors associated with severe dengue

In the multivariable analysis, higher serum 25(OH)D concentration remained associated with lower odds of severe dengue after adjustment for age group, sex, and IgG seropositivity (adjusted odds ratio = 0.921 per 1 ng/mL increase; 95% confidence interval 0.855-0.992; *P* = 0.029). Specifically, each 1 ng/mL increase in serum 25(OH)D concentration was associated with approximately 7.9% lower odds of severe dengue. The persistence of this association after multivariable adjustment indicates that it was not fully explained by the measured demographic and serological factors included in the model; however, residual confounding from other host, environmental, or disease-related factors remains possible.

In contrast, age group and IgG seropositivity were no longer statistically significant after multivariable adjustment, and sex was also not statistically associated with severe dengue in the adjusted model. The attenuation of the associations for age group and IgG seropositivity may reflect the complex interrelationships among age, previous dengue exposure, host immune responses, and disease severity. The persistence of the association between serum 25(OH)D concentration and severe dengue after multivariable adjustment supports further investigation of vitamin D status as a potential host-related factor in dengue severity [11,17]. Similar associations between lower vitamin D concentrations and adverse outcomes have also been reported in sepsis and other acute inflammatory conditions [29].

Nevertheless, the cross-sectional design precludes determination of temporal relationships. It remains unclear whether lower vitamin D concentrations predisposed children to severe dengue or were themselves influenced by the acute inflammatory response accompanying severe disease. Prospective longitudinal studies with serial vitamin D measurements before and during infection are needed to clarify causality.

### Clinical and research implications

The present findings suggest that circulating 25(OH)D concentration may represent a host-related factor associated with dengue severity in children. However, because serum 25(OH)D was measured during acute illness and the study was cross-sectional, the observed association cannot establish whether lower vitamin D concentrations preceded severe disease or resulted partly from the acute inflammatory response. Therefore, these findings should not be interpreted as evidence that vitamin D supplementation prevents or treats severe dengue. Rather, they provide a rationale for prospective studies incorporating serial vitamin D measurements and more comprehensive assessment of host, environmental, virological, and immunological factors. If confirmed in larger prospective cohorts, vitamin D status may contribute to a better understanding of host determinants of dengue severity and provide a basis for future mechanistic and interventional research.

## Limitations

Some limitations should be acknowledged. First, the cross-sectional design and measurement of serum 25(OH)D during the critical phase of illness preclude determination of temporality or causality. Because 25(OH)D may behave as a negative acute-phase reactant, the observed lower concentrations in severe dengue may reflect the acute inflammatory response rather than pre-existing vitamin D status. Second, the single-center design and the relatively small number of severe dengue cases (*n* = 32) limited statistical power and generalizability, particularly for analyses of polymorphisms with low minor allele frequencies. Third, the nominal deviations from Hardy-Weinberg equilibrium observed for FokI and ApaI require cautious interpretation and may reflect sampling variation, population structure, or genotyping-related factors. Fourth, although the multivariable model adjusted for age group, sex, and IgG seropositivity, residual confounding cannot be excluded. Potentially relevant factors such as nutritional status, sunlight exposure, seasonal variation, timing of blood sampling during the illness course, and previous dengue exposure were not fully accounted for. Therefore, the adjusted association between serum 25(OH)D concentration and severe dengue should not be interpreted as evidence of an independent causal effect. Larger prospective multicenter studies with serial vitamin D measurements, more comprehensive assessment of potential confounders, and adequately powered haplotype-based genetic analyses are needed to validate these findings.

## Conclusion

In this cross-sectional study of Vietnamese children hospitalized with dengue, lower serum 25(OH)D concentrations were associated with severe dengue after adjustment for age group, sex, and IgG seropositivity and correlated with platelet count and hematocrit. No significant associations were detected between the examined VDR polymorphisms and either dengue severity or serum 25(OH)D concentrations. These findings support further investigation of vitamin D status as a host-related factor associated with dengue severity but do not establish a causal relationship. Larger prospective multicenter studies with serial vitamin D measurements and more comprehensive assessment of potential confounders are needed to validate these findings and clarify temporality and causality.

## Ethical approval

The study was conducted in accordance with the Declaration of Helsinki. The study protocol was approved by the Biomedical Research Ethics Committee of Can Tho University of Medicine and Pharmacy (Approval No. 24.093.GV/PCT-HĐĐĐ; November 16, 2024). Administrative permission was obtained from Ca Mau Obstetrics and Pediatrics Hospital. Written informed consent was obtained from the parents or legal guardians of all participants before enrollment.

### Informed consent and patient details

Written informed consent to take part in the study and to publish the article has been obtained from all participants or their legal representatives. The privacy rights of participants have been observed.

### Human and biological material

This study included organ or tissue donors. This study includes human biological material and consent was obtained by donors, or their next of kin or legal representatives, for use in this study and for publication of the article. The samples used in this research were not sourced from executed prisoners or prisoners of conscience.

### Studies in human

This study was performed in compliance with relevant laws, regulatory frameworks and guidelines where the research took place. This study was approved by the Biomedical Research Ethics Committee of Can Tho University of Medicine and Pharmacy (Approval No. 24.093.GV/PCT-HĐĐĐ).

## CRediT authorship contribution statement

**Phan Viet Hung:** Writing – review & editing, Writing – original draft, Methodology, Investigation, Formal analysis, Data curation, Conceptualization. **Nguyen Minh Phuong:** Writing – review & editing, Writing – original draft, Methodology, Investigation, Formal analysis, Data curation, Conceptualization. **Nguyen Thi Cam Tien:** Writing – review & editing, Methodology, Investigation, Data curation. **Tran Thi Huynh Nhu:** Writing – review & editing, Investigation, Data curation. **Pham Minh Quan:** Writing – review & editing, Investigation, Data curation. **Truong Van Dat:** Writing – review & editing, Resources, Investigation, Data curation. **Nguyen Thi Thuy Duong:** Writing – review & editing, Resources, Investigation, Data curation. **Thai Minh Dien:** Writing – review & editing, Methodology, Formal analysis, Data curation. **Trinh Nhut Truong:** Writing – review & editing, Resources, Investigation, Data curation. **Hua Kim Chi:** Writing – review & editing, Resources, Investigation, Data curation. **Bui Kim Dang:** Writing – review & editing, Resources, Investigation, Data curation. **Vo Van Thi:** Writing – review & editing, Supervision, Project administration, Methodology, Conceptualization.

## Declaration of competing interest

The authors have no competing interests to declare.

## Appendix Supplementary materials

Image, application 1
