# Supplementary material for: Associations of serum 25-hydroxyvitamin D and vitamin D receptor polymorphisms with dengue severity in hospitalized Vietnamese children
Source: IJID Reg. 2026 Aug 30;21:100989. doi: 10.1016/j.ijregi.2026.100989 (PMC13594944; doi:10.1016/j.ijregi.2026.100989)
Supplement: Supplementary file 1 [file mmc1.docx]

Supplementary Material

Associations of Serum 25-Hydroxyvitamin D and Vitamin D Receptor
Polymorphisms with Dengue Severity in Hospitalized Vietnamese Children

**Supplementary Table S1.** Correlations between serum 25-hydroxyvitamin D concentrations and laboratory parameters

| **Laboratory parameter** | **Correlation coefficient (r)** | **p-value** |
| --- | --- | --- |
| Platelet count (minimum) | 0.201 | 0.005 |
| Hematocrit (maximum) | -0.206 | 0.004 |
| White blood cell count (minimum) | 0.132 | 0.069 |

*Spearman's rank correlation coefficient (r) is reported. A p-value < 0.05 was considered statistically significant.*

**Supplementary Table S2.** Hardy–Weinberg equilibrium analysis of vitamin D receptor polymorphisms

| **Polymorphism** | **χ²** | **HWE p-value** |
| --- | --- | --- |
| FokI | 4.215 | 0.040 |
| BsmI | 0.730 | 0.393 |
| ApaI | 3.953 | 0.047 |
| TaqI | 0.318 | 0.573 |

FokI and ApaI showed slight deviations from Hardy–Weinberg equilibrium, whereas BsmI and TaqI were consistent with Hardy–Weinberg equilibrium.

**Supplementary Table S3.** Serum 25-hydroxyvitamin D concentrations according to vitamin D receptor genotype

| **SNP** | **Genotype** | **n** | **Vitamin D (ng/mL) Median (IQR)** | **p-value** |
| --- | --- | --- | --- | --- |
| *FokI (rs2228570)* | CC | 69 | 26.73 (22.28–31.37) | 0.124 |
|  | TC | 76 | 23.78 (20.63–29.17) |  |
|  | TT | 39 | 25.00 (21.45–28.68) |  |
| *BsmI (rs1544410)* | GG | 165 | 25.16 (21.18–29.76) | 0.880 |
|  | GA | 22 | 26.54 (19.97–30.75) |  |
| *ApaI (rs7975232)* | GG | 85 | 25.34 (21.62–28.75) | 0.561 |
|  | GT | 76 | 26.82 (21.21–31.05) |  |
|  | TT | 7 | 26.64 (19.53–31.20) |  |
| *TaqI (rs731236)* | TT | 154 | 25.84 (21.26–29.87) | 0.556 |
|  | TC | 14 | 27.27 (22.76–30.75) |  |

*Data are presented as median (interquartile range). Comparisons were performed using the Kruskal–Wallis test for three genotype groups and the Mann–Whitney U test for two genotype groups.*
